# Unsupervised model for structure segmentation applied to brain computed tomography

**DOI:** 10.1371/journal.pone.0304017

**Published:** 2024-06-13

**Authors:** Paulo Victor dos Santos, Marcella Scoczynski Ribeiro Martins, Solange Amorim Nogueira, Cristhiane Gonçalves, Rafael Maffei Loureiro, Wesley Pacheco Calixto

**Affiliations:** 1 Electrical, Mechanical & Computer Engineering School, Federal University of Goias, Goiania, Brazil; 2 Department of Radiology, Hospital Israelita Albert Einstein, Sao Paulo, Sao Paulo, Brazil; 3 Federal University of Technology - Parana, Ponta Grossa, Parana, Brazil; 4 Technology Research and Development Center (GCITE), Federal Institute of Goias, Goiania, Brazil; Yarmouk University, JORDAN

## Abstract

This article presents an unsupervised method for segmenting brain computed tomography scans. The proposed methodology involves image feature extraction and application of similarity and continuity constraints to generate segmentation maps of the anatomical head structures. Specifically designed for real-world datasets, this approach applies a spatial continuity scoring function tailored to the desired number of structures. The primary objective is to assist medical experts in diagnosis by identifying regions with specific abnormalities. Results indicate a simplified and accessible solution, reducing computational effort, training time, and financial costs. Moreover, the method presents potential for expediting the interpretation of abnormal scans, thereby impacting clinical practice. This proposed approach might serve as a practical tool for segmenting brain computed tomography scans, and make a significant contribution to the analysis of medical images in both research and clinical settings.

## Introduction

Neurological diseases pose a significant risk not only to individual development but also to overall productivity. Currently, some of the most prevalent neurological disorders, such as dementia, stroke, epilepsy, Parkinson’s disease, multiple sclerosis, migraines, and tension-type headaches, generate an estimated economic cost of nearly $789 billion in the United States [1]. These disorders involve congenital, developmental, or acquired abnormalities that affect the brain, spinal cord, and nerves [2].

The World Health Organization (WHO) estimates that one in three people will be affected by neurological disorders in their lifetime. These disorders are the leading cause of disability and the second leading cause of death, resulting in more than six million deaths each year. Given these statistics, public health and society must prioritize the understanding and effective treatment of neurological disorders. Prevention strategies, early diagnosis, and effective treatments are critical to minimizing the negative impact of these conditions on patients’ health while reducing the associated economic burden [3].

The high prevalence of neurological diseases and the growing number of neuroimaging studies stored in repositories [4, 5] emphasize the importance of using artificial intelligence (AI) to develop models that can assist physicians in early diagnosis, thus improving patient care [6, 7]. By analyzing vast amounts of data, AI can identify intricate and subtle patterns that humans cannot observe.

AI has greatly advanced in the analysis of medical images, particularly through the process of annotation. This involves trained radiologists identifying and labeling specific areas with anatomical structures, lesions, or other significant features, which is crucial for AI algorithms involved in tasks such as detection, segmentation, and classification [8, 9]. The use of well-organized medical image datasets with accurate labeling facilitates the development of computational technologies for efficient classification and preliminary medical diagnoses in a supervised manner [10]. Supervised machine learning algorithms are trained using labeled data, establishing associations and inferences between data and relevant categories. This supervised approach allows the generalization and inference of new tests based on previously learned patterns. Among various supervised learning methods, artificial neural networks (ANNs) have emerged as a particularly promising approach [11, 12]. However, the annotation process can be resource-intensive in terms of time and finances. Moreover, available datasets often lack adequate annotations or a satisfactory quantity for the effective training of AI models [13–15].

Segmentation, the division of the image into regions, objects, or pixels, with the aim of separating the objects of interest from other elements [16], is a technique frequently applied, often performed by ANNs [17], and is important in biomedical applications, particularly for delineating anatomical structures [18]. In neuroimaging, the segmentation of intracranial structures facilitates the visualization and classification of brain tissues, aiding in the identification of abnormalities, including tumor localization [19–21]. Deep Neural Networks (DNNs), a subtype of neural networks, have proven effective for segmentation, acting as supervised classifiers and achieving more accurate results [18, 22, 23].

Numerous studies have employed supervised deep learning techniques for segmentation purposes. Monteiro *et al.* proposed the use of supervised deep learning to quantify brain injuries using the CQ500 dataset [24, 25]. Ronneberger, Fischer, and Brox, as well as Li *et al.*, developed a specialized deep neural network architecture called U-Net for brain hemorrhage segmentation, achieving an impressive 98% accuracy in identifying bleeds across two distinct datasets [26, 27]. However, it is essential to acknowledge that these approaches are limited by the availability of pre-annotated datasets. Segmentation of brain images is challenging due to image noise, especially for computed tomography (CT) images [28]. Additionally, supervised segmentation involves certain considerations: i) it can be costly as it relies on annotations from experts with potential interobserver variability, ii) modeling different exam types incurs high computational costs as models need to be trained for each exam type. and iii) the CT acquisition protocol may vary from scanner to scanner, differing in terms of signal to noise ratio (SNR), contrast, slice thickness, and spatial resolution, among others.

On the other hand, unsupervised segmentation methods have also attracted attention. These methods offer unique advantages since they do not rely on pre-labeled data and can effectively deal with image noise. Balafar *et al*. [28] provided a comprehensive review of supervised and unsupervised segmentation techniques and suggested further research to enhance the speed, accuracy, and integration of these methods. A notable unsupervised segmentation approach was proposed by Atkins & Mackiewich [29], who used anisotropic filters and image processing techniques, such as noise removal, to perform unsupervised segmentation of brain lesions and generate brain contour masks. This automated method demonstrated the ability to segment images obtained from different scanners and resolutions, overcoming the limitations associated with data heterogeneity. Their work contributed to unsupervised segmentation in neuroimaging, and their results highlighted the potential to advance the field in terms of accuracy and applicability.

Lee *et al*. [30] proposed an approach that combines classic clustering algorithms, such as *k*-means and fuzzy *c*-means, for the segmentation of brain CT images into three distinct regions. In their study, the authors employed decision trees to analyze the interrelation between components in normal and abnormal regions within brain CT images. These unsupervised techniques have shown promising results in medical image segmentation, effectively addressing challenges associated with the need for specialized annotations and the presence of image noise. As a result, they enhanced the accuracy and reliability of medical image segmentation techniques, pushing the boundaries of the field and creating new opportunities for improved diagnosis.

Recent studies have explored unsupervised segmentation for brain images acquired through magnetic resonance imaging (MRI). For example, Dalca *et al*. [31] proposed a novel approach that combines Bayesian inference with classical probabilistic segmentation based on brain atlases, incorporating deep learning techniques. The authors developed neural networks that can detect, delineate, and recognize abnormalities in brain images, allowing the segmentation model to be trained on new MRI scans without manual annotation. Experimental results showed that the method enables accurate segmentation regardless of MRI contrast.

Mahata *et al*. [20] proposed a fuzzy segmentation technique for brain MRI. Their method consists of combining the Gaussian function with local contextual information to establish associations between neighboring pixels. Segmentation is performed by using the Gaussian function to estimate the heterogeneous intensity within each tissue region using the local gradients of the image. The authors conducted simulations using two databases of brain MRI scans and demonstrated that the proposed method is efficient compared to other clustering algorithms based on fuzzy logic. However, it is fundamental to note that their proposed approach is specifically tailored to MRI studies.

Khan *et al*. [32] proposed a method for accurate classification of brain tumors in MRI images. Their method comprised three phases: i) preprocessing, ii) segmentation of brain tumor using the *k*-means clustering technique, and iii) classification of tumors as benign or malignant. To refine the accuracy of the classification process, the authors introduced the concept of synthetic data augmentation. In this technique, additional data is generated to expand the training dataset considered by the classifier. The approach was evaluated using the BraTS2015 datasets, and results show the robustness of the proposed strategy, particularly emphasizing the effectiveness of clustering as a segmentation technique.

Raja *et al*. [33] presented a methodology to classify brain tumors in MRI scans by introducing a hybrid deep autoencoder with a Bayesian fuzzy clustering segmentation approach. Following segmentation, they extracted information metrics using dispersion transform and entropy methods. This work is characterized by the integration of unsupervised and supervised approaches, although it is limited to MRI scans. In a similar context, Hua *et al*. [34] proposed a clustering technique based on fuzzy *c*-means to improve the accuracy of segmentation in brain MRI scans. Their method incorporates a visualization mechanism with adaptive weight learning, which assigns an optimal weight to each visualization based on its cluster contribution. The segmentation result was obtained by combining these visualizations. Compared to other clustering algorithms, the authors’ method exhibited superior adaptability and performance.

The segmentation of medical images using MRI has been extensively explored in the literature, often combining both supervised and unsupervised learning techniques. According to Lenchik *et al*. [35], the brain segmentation studies conducted in 2019 indicate a predominant use of MRI over CT. In Brazil, there are approximately 15.6 CT machines per million inhabitants, with MRI machines generally having higher maintenance and depreciation costs [36, 37]. As a result, the widespread availability and rapid imaging acquisition of CT scans justify their frequent use, especially in emergency cases such as stroke, where prompt diagnosis is crucial for effective treatment.

Table 1 presents a chronological overview of studies that have addressed MRI and CT image segmentation, including the study by Kim, Kanezaki, and Tanaka [38]. Their work proposes an unsupervised segmentation approach applicable to various image types beyond the medical domain. The method uses a deep neural network architecture with different filters and processes to group similar features, eliminating the need for training or manual labeling. However, adapting this particular method for CT scans is crucial considering the unique characteristics of this imaging modality.

**Table 1:** Summary of studies addressing the segmentation of magnetic resonance imaging and computed tomography images.

Approach/Method	Reference	Context/Description
Unsupervised segmentation	Lee *et al*. [30]	Brain CT images with abnormality extraction approach.
Supervised segmentation and clustering	Mahata *et al*. [20]	Brain MRI images with estimation of heterogeneous intensity using fuzzy clustering algorithm induced by local contextual information and Gaussian function.
Unsupervised segmentation	Dalca *et al*. [31]	Brain MRI images based on Bayesian approach.
Supervised segmentation, classification, and clustering	Raja *et al*. [33]	Brain tumor MRI images using hybrid deep autoencoder with Bayesian fuzzy clustering approach.
Unsupervised segmentation and clustering	Kim, Kanezaki & Tanaka [38]	Any image based on differentiable feature clustering.
Supervised segmentation, classification, and clustering	Khan *et al*. [32]	Brain tumor MRI images using *k*-means clustering algorithm, deep learning, and synthetic data.
Supervised segmentation and clustering	Hua *et al*. [34]	Brain MRI images using fuzzy *c*-means clustering algorithm with multiple views.

To bridge this gap, this work aims to adapt the method of Kim, Kanezaki, & Tanaka [38] for CT scan segmentation using a predetermined number of masks and performing an initial calibration of the neural network with reference images. This approach allows the segmentation of similar images without training or manual annotation.

The central hypothesis of this study is as follows: if it is possible to develop an unsupervised segmentation model that uses an end-to-end approach to segment intracranial structures and optimizes the network’s hyperparameters, then it can be effectively applied to the specific context of CT exams. This approach would eliminate the need for expert conceptual changes, reduce computational costs, and simplify the annotation task by facilitating the identification of regions with abnormalities. The main objective is to implement a deep neural network architecture for segmenting intracranial structures without prior labeling, manual annotation, or supervision. Specific objectives include: i) evaluating different training techniques for the neural network, ii) applying the optimization process to determine optimal values for the network’s hyperparameters, iii) controlling the number of masks used in the segmentation process, and iv) evaluating the performance of the proposed approach through validation with domain experts and a comparative analysis with other studies in the literature.

This paper presents an original unsupervised method for segmenting medical images, characterized by its high flexibility in defining the number of masks and the inclusion of customizable metrics for each segment. The originality and innovation of this approach lie in optimizing the network’s hyperparameters, without relying on pre-existing labels. This strategy is designed to reduce the financial and time costs, thus enabling faster and more efficient diagnoses. A key significance of this method lies in the effort to simplify traditional supervised segmentation models, which typically demand manual annotation and extensive computational resources. Moreover, the proposed model shows promising potential for application in several medical fields.

This article is structured as follows: The Theoretical Background section discusses the conceptual foundations, which include important theories such as brain anatomy, the Hounsfield Scale, and window sliding. The Methodology section provides a detailed description of the proposed approach, while Results section describes the experiments conducted and highlights results, complemented by relevant discussions. Finally, the Conclusion section succinctly summarizes the main conclusions and contributions of this research.

## Theoretical background

This section provides an overview of the anatomy, structure, and function of the brain. It also discusses the main differences between CT and MRI and the methods of annotating and labeling medical images. In addition, it explains the concept of segmentation techniques, focusing on the unsupervised approach for medical images.

### Brain structures

The human brain consists of several interconnected structures that are involved in higher cognitive functions, memory formation, sensory processing, autonomic and endocrine regulation, motor coordination, and vital functions [39, 40]. It comprises the cerebrum, diencephalon, brainstem (midbrain, pons, and medulla), and cerebellum. The cerebrum, the largest part of the brain, encompasses the cerebral hemispheres, basal ganglia, and white matter tracts. Each cerebral hemisphere is divided into five lobes listed in descending order of size: the frontal lobe, temporal lobe, parietal lobe, occipital lobe, and insular lobe. The corpus callosum serves as the connection between the two hemispheres. The brain has characteristic folds called gyri (singular: gyrus) and grooves called sulci (singular: sulcus), which increase its surface area for information processing [41–43].

**Fig 1:**
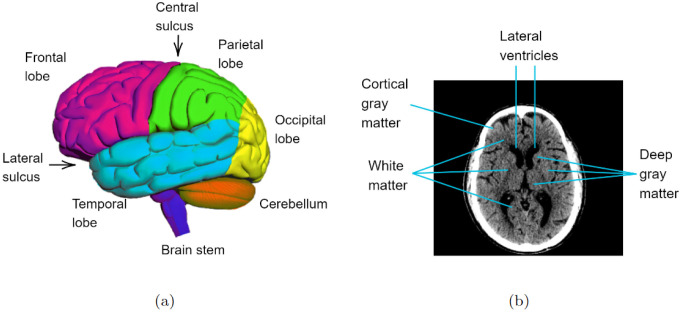
Brain mapping: (a) specific brain regions and (b) different brain structures.

Fig 1(a), adapted from [44], illustrates the brain’s surface. The gray matter is the substance of the brain that contains the neuronal cell bodies. Within the cerebrum, the two main grey matter locations are on the surface of the gyri, known as the cortical grey matter, and in the nuclei of the basal ganglia. In contrast, white matter consists of fiber tracts comprising neuronal axons [45]. Compared to white matter, gray matter has a higher density, enabling their differentiation on imaging examinations [46]. The brain also has a ventricular system consisting of internal ventricles filled with cerebrospinal fluid (CSF): two lateral ventricles, a third ventricle in the midline, and a fourth ventricle [39]. This fourth ventricle, located between the pons of the brainstem and the cerebellum, is mainly visible in lower cross-sectional slices [47–51].

The segmentation of brain structures contributes to quantifying brain volume and diagnosing neurological diseases [39, 40]. The Fig 1(b) illustrates an axial image of a brain CT scan, highlighting discernible regions.

### Computed tomography × magnetic resonance imaging

**Fig 2:**
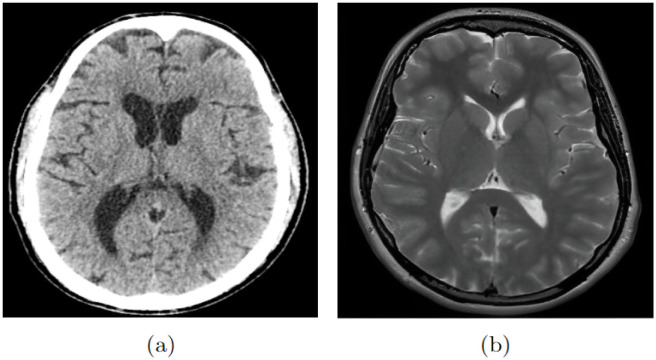
Brain exams: (a) computed tomography e (b) magnetic resonance.

CT and MRI are widely used imaging techniques for the diagnosis of neurological diseases. CT uses X-rays to produce detailed cross-sectional images of the human body, while MRI uses strong magnetic fields and radiofrequency waves to produce images with higher contrast resolution, allowing better differentiation between various tissue types based on their magnetic properties [52]. In contrast to CT, MRI is not associated with ionizing radiation, which makes it safer in terms of radiation exposure. However, CT has distinct advantages, such as higher spatial resolution, faster image acquisition, and lower costs, making it more accessible in public institutions [52]. Fig 2(a) and 2(b), adapted from Le [53], illustrates the differences between a CT and an MRI scan performed on the same individual and in the same cross-sectional view of the brain.

In CT, the X-rays pass through the body and are captured by sensors that convert them into electrical signals. These signals are reconstructed by a computer, resulting in volumetric images presented in slices as the X-ray tube completes a full 360° rotation around the body [52]. Modern scanners typically produce images with an average thickness of 0.5*mm*. Ongoing advances in medical imaging and technology have led to specific protocols designed for accurate diagnoses. These protocols seamlessly integrate devices, standardized procedures, and AI applications [54]. To ensure interoperability and consistency, digital images adhere to standardized formats, such as the DICOM standard (Digital Imaging and Communications in Medicine). DICOM defines universal standards for storage and communication, regardless of the manufacturer or vendor [55]. This standardization improves compatibility and facilitates the seamless exchange of medical imaging data in the healthcare ecosystem.

In CT scans, the digital image is represented by intensity values expressed with Hounsfield units (HU), a numerical scale that assigns values to different substances and tissues based on their radiological attenuation [56]. Hounsfield units are derived from a linear transformation of the measured attenuation coefficients. This transformation is based on the radiodensities of air (assigned as 0 HU) and pure water (assigned as −1000 HU) at standard temperature and pressure. Each pixel in the CT image is assigned an intensity value in HU [57].

This approach enables the classification of different tissues, with bones appearing as white regions and soft tissues such as muscles and organs having intermediate values visualized in shades of gray [56]. Regions containing air are displayed in darker tones. By applying filters or thresholds, specific tissues can be highlighted for visualization. Generally, CT images use 12 − *bit* images capable of storing values between −1024 and 3071 HU [58]. The presentation of these values is determined by the application of specific window and level settings, allowing for better visualization of anatomical structures in different parts of the human body [56, 59, 60]. This windowing adjusts the values in the grayscale range in radiological exams, improving the visualization of structures of interest. It involves selecting specific attenuation intervals and associating them with corresponding ranges in the grayscale. The proper use of this procedure results in improvements in identifying the regions [61].

### Annotation and labeling in medical images

The manual annotation process of digital images is performed by experts who identify and label spatial regions present in the image, providing descriptions and comments in the textual form [62]. In the medical field, image annotation requires precision, often involving the participation of multiple radiologists who perform independent annotations [54]. After individual labeling, the annotations are collectively reviewed for further corrections and updates. However, this manual annotation process is financially and temporally demanding, often requiring the involvement of multiple professionals [62].

To address these challenges, several computational tools have been developed to enable automatic image annotation, ranging from classification labels to pixel-by-pixel segmentation [63]. In medical images, automatic annotation is applied to label MRI and CT images for training machine learning and deep learning models [54]. These tools play a fundamental role in the segmentation of structures in several organs, such as breast masses, lung nodules, retinal vessels, and tumors in the liver, brain, and other regions [62].

### Unsupervised segmentation

Image segmentation can be performed in two ways: i) supervised and ii) unsupervised. In supervised segmentation, the model is trained with labeled data, where labels and annotations are input information about the regions of interest in the image [64, 65]. In contrast, in unsupervised segmentation, the model is trained without labels, using clustering techniques or pixel comparisons to automatically identify regions or objects in the image. The supervised approach provides more accurate results but requires prior annotation, while the unsupervised approach is useful when labels are not available or when the image has an unknown variety of regions [66].

Automatic segmentation of medical images is a growing area of research as it promises to aid the diagnosis and monitoring of patients with various diseases [66]. With the integration of AI, image analysis tasks have become more accessible, demanding higher precision in the delineation of regions of interest. The main goal of segmentation is to simplify the representation of relevant information by dividing the image into different regions using techniques that pinpoint objects or their boundaries. There are different approaches, including region-based, edge-based, or object boundary-based techniques [67]. Within this spectrum, unsupervised segmentation aims to separate objects from the image background through pixel comparison or grouping based on similarities. This process involves the application of calculations and evaluation functions tailored to the specific context.

The unsupervised DNN approach has been widely employed in segmenting brain structures or lesions, combining statistical methods, comparative methods, and clustering to achieve accurate results [66]. Recent studies have applied these methods in MRI, exploiting the high quality and contrast resolution of these images to delineate objects and regions in the brain [31, 68, 69]. Evaluation metrics are applied to measure the AI model’s ability to make correct or incorrect predictions, usually based on the confusion matrix. The model’s performance is assessed by comparing true positives to the desired values, resulting in a positive measure for the highest number of correct predictions [70].

The loss function is fundamental for quantifying the difference between the output of the model and the desired value, representing errors on a per-observation basis. Unlike the evaluation function, which includes all model data, the loss function measures the errors individually [71]. In unsupervised approaches, where there are no expected values and no labeled data for training, the loss or the evaluation function serves as a reference during the training of the feature extraction model. The applied function indicates the desired behavior, taking into account errors in prediction or model performance.

### Optimization process

The optimization process of DNN models occurs during the training phase. In this process, the model is adjusted via its hyperparameters. These are configurations and decisions that influence the behavior and efficiency of the model but are not learned from the data during training [72]. Some of the optimizers frequently used in DNN are Adaptive Moment Estimation (Adam), Root Mean Square Propagation (RMSprop), and Stochastic Gradient Descent (SGD) [73]. Adam integrates elements of algorithms such as RMSprop [74], while RMSprop is a variant of SGD that adjusts the learning rate individually for each parameter [75]. SGD is the effective version of the classic gradient descent algorithm and is particularly suitable for large datasets [76].

The main goal of most optimization processes in DNNs is to minimize the loss function. This is achieved by strategies that search for optimal values for hyperparameters [77]. Optuna is a hyperparameter optimization library for DNNs that uses the history of experiments to determine which hyperparameter configurations are best suited for a given problem [78]. Optuna employs techniques such as decision trees and Bayesian optimization to explore the hyperparameter search space and determine the most efficient combination. It uses the Parzen density estimator (PDE) to model the probability distribution of the hyperparameters during the optimization process [73, 79]. In addition, Optuna interacts with applications or platforms via an application programming interface (API), which enables communication between different software components.

## Methodology

Computed tomography (CT) is a necessary diagnostic tool in the detection of various neurological diseases. However, careful annotation of the images can be challenging, requiring specialized professionals to identify and annotate different brain structures in the search for abnormalities. Therefore, an approach is proposed that employs automated brain region segmentation techniques to expedite the annotation process and minimize manual intervention. Thus, a deep neural network (DNN) is implemented to segment intracranial structures without relying on pre-existing labeling, manual annotation, or direct supervision. Additionally, different training techniques for neural networks are explored, the network hyperparameters are optimized, the ideal number of masks for segmentation is determined, and the overall performance of the approach is evaluated. Segmentation results are validated by comparing them with expert opinions in the field and contextualizing them with similar studies from the literature. The routines utilized in this paper are made available at Santos *et al*. [80].

## Foundation and criteria of the methodology

The proposed methodology focuses on image segmentation without the need for pre-specified training images or pixel labels. In this approach, once the target image is provided, pixel labels and feature representations are jointly optimized, and their parameters are updated through gradient descent. The proposed process alternates between predicting labels and learning network parameters to satisfy three main criteria: i) pixels with similar characteristics should be assigned the same label, ii) spatially contiguous pixels should be assigned the same label, and iii) the number of unique cluster labels should contain a high number of pixels. These criteria reflect the intuition that desired image segmentation groups similar pixels forming parts or salient objects in the image while maintaining spatial continuity and distinguishing between distinct patterns. To meet these criteria, the approach minimizes the combination of similarity and spatial continuity losses.

**Fig 3:**
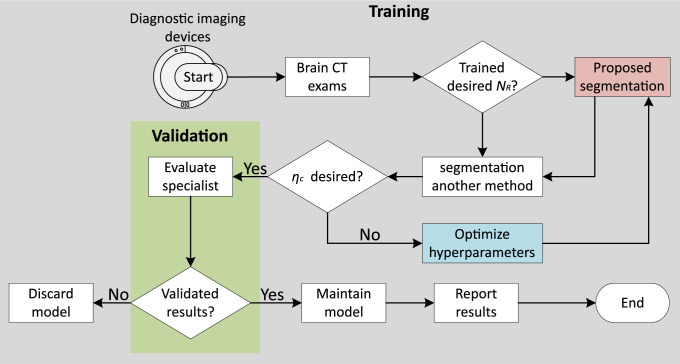
Flowchart of the proposed methodology.

The study introduces a novel end-to-end network architecture for unsupervised segmentation of intracranial medical images, which includes normalization and a differentiable clustering function. Furthermore, the spatial continuity loss function has its parameters optimized to mitigate limitations related to fixed segment boundaries, as observed in previous works [38]. The proposed architecture employs linear classification to categorize the features of each pixel into classes, followed by normalization and classification to determine cluster labels. The similarity loss of features is calculated based on the cross-entropy between the normalized response maps and the predicted cluster labels, while the spatial continuity loss is based on the norm-*L*_*p*_ of horizontal and vertical differences in the response map [81]. The evaluation and validation flow of the proposed methodology is illustrated in Fig 3.

The flowchart shown in Fig 3 outlines the methodology for segmenting brain CT exams. The process starts with the selection of a database of brain CT scans to obtain the desired number of labels *N*_*R*_. It is checked whether there is a pre-trained model that can generate the desired labels. If no such model exists, a new model is trained (Training). The images segmented by the proposed method are compared with the images generated by existing tools such as CTSeg [82, 83] using segmentation evaluation metrics (Validation). If results of these metrics are in the predefined acceptable threshold region*η*_*c*_, they are evaluated by experts. If there is disagreement, the model optimization process is repeated until results reach the desired *η*_*c*_. If the segmentation is approved by the experts, results are presented in text form. Otherwise, the model is discarded.

### Data pre-processing

In order to use CT scans with DNNs, the data must be standardized. To this end, it must be ensured that all exams in the dataset have the same number of slices and uniform dimensions. To achieve this standardization, the exams are subjected to spatial dimension interpolation. In this process, pixels are created or eliminated based on neighboring pixels, allowing for convenient spatial image resizing [84, 85]. Result is a predefined number of image slices and a uniform resolution in width and height. This standardization is necessary to ensure that the deep neural network can analyze the data accurately.

To eliminate irrelevant information in exams, a windowing procedure is applied with values of 40*HU* for the window center and 80*HU* for the window width, according to standard medical practice [86, 87]. An algorithm is then used to map the Hounsfield scale to a new range of gray tones. This approach simplifies the calculations during the training of the neural networks, as it involves numbers on a reduced scale, making the process more efficient [60]. At the end of the preprocessing phase, the data structure is obtained with the dimensions [*N*_*fatia*_ *timesL timesA*], where *N*_*fatia*_ is the number of slices, *L* is the width and *A* is the height of the image in pixels.

### Architecture of the proposed segmentation model

**Fig 4:**
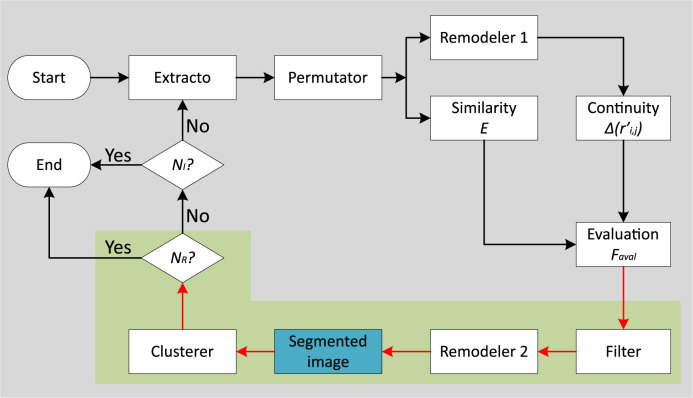
Flowchart of the proposed architecture.

Since we use the segmentation model developed by Kim, Kanezaki, and Tanaka [38], in which the authors follow an unsupervised approach that uses clustering techniques and metrics to evaluate static similarity *S* and continuity rate Δ(*r*′ *i*, *j*), we propose a different approach using dynamic values to adjust the calculation of *S* and Δ(*r*′ *i*, *j*), based on the desired *N*_*R*_ as the output of segmentation of CT scans. The architecture of the proposed segmentation model is indicated in the gray block in Fig 3 and illustrated in Fig 4, where the black flow represents the learning phase of the segmentation, while the red flow represents the final phase of the iterative process when the desired *N*_*R*_ is achieved.

The feature extractor, as shown in Fig 4, configurable convolutional layers and activation functions that are important for extracting features from images, especially in unsupervised approaches that aim to address the similarity constraint between pixels [88, 89]. After feature extraction, normalization is performed with a certain number of convolutional filters *N*_*F*_ for each layer [90]. Sivakumar [91] describes that CT images are inherently susceptible to noise and that grouping pixels based on *S* requires normalization of the pixels in the image. In the context of this study, images with dimensions [*L* × *A*] pixels are processed by the neural network, resulting in a one-dimensional tensor [*P*_*n*_]. Each position in this structure stores a processed image while maintaining the original dimensions [*L* × *A*]. The result of the normalization is then a three-dimensional structure with the dimensions [*L* × *A* × *P*_*n*_], which consists of images processed by different *N*_*F*_.

The permutator works with the output of the feature extractor, which consists of constructing the tensor [*P*_*n*_]. This structure provides data for the evaluation process of segmentation learning, shown in the black flow in Fig 4, and also feeds the necessary structure for the application of the one-dimensional maximum value filter, represented by the red flow in Fig 4. Each element of [*P*_*n*_] corresponds to an image processed by a specific *N*_*F*_. Thus, considering *N* as the number of pixels in each image, this data structure is transformed into a new tensor with dimensions [*N* × *P*_*n*_] in pixels. After permutation, two reconfiguration procedures are performed: i) Remodeler 1 and ii) Remodeler 2. The aim of these procedures is to reconstruct the data structure in the corresponding dimensions. Remodeler 1 processes the data structure of the permutator, which is scaled to [*N* × *P*_*n*_], resulting in a new structure with the dimensions [*A* × *L* × *P*_*n*_]. On the other hand, Remodeler 2 considers the tensor [*N*] with input elements and converts this information into the tensor [*A* × *L*] pixels.

The calculation of *S* generates positive values for similar features, while negative values are always converted to zero. This approach adjusts the values to the desired range, creating the similarity map. This map ensures that similar features are related or close to each other while distinguishing different behaviors depending on the type of image or examination processed. In this work, the calculation of *S* is done using the cross-entropy function, given by:

$$\begin{array}{c} E=-\sum_{i=1}^{N}p\left(x_{i}\right)\cdot \text{log}\left[q\left(x_{i}\right)\right] \end{array}$$ (1)

in which *p*(*x*_*i*_) denotes the probability of the actual class obtained from the reference map for pixel *x*_*i*_, and *q*(*x*_*i*_) represents the probability estimated by the neural network from the similarity map for the same pixel. The cross entropy serves as an indicator of the agreement between the model output and the reference data. In medical image segmentation, cross entropy is the metric used to evaluate the correspondence between the segmentation determined by the neural network and the desired segmentation, allowing the quality of the segmentation obtained to be quantified.

The calculation of Δ(*r*′ *i*, *j*) is used to accurately identify objects present in images. This process is based on the observation that similar pixel values indicate continuity in certain regions, while abrupt changes may indicate the boundaries of regions or objects. Δ(*r*′ *i*, *j*) analyzes the three-dimensional tensor [*A* × *L* × *P*_*n*_] by comparing all its elements. For example, if a part of the image is contained in a certain element, the technique calculates the difference of possible reliefs between other elements and thus measures the continuity of these features. The result of $\Delta (r_{i,j}^{\prime })$ is the delineation of the regions present in the images, which indicates the differences between the types of objects present.

The $\Delta (r_{i,j}^{\prime })$ can be computed by overlapping filtered images on the original image and shifting them in different directions relative to the original image. The pixel at a particular position appears displaced in the overlapping images, allowing its distance to the same pixel in the other images to be calculated. Smaller distance values indicate spatial continuity, which means that there were no abrupt changes in the object. Larger distance values, on the other hand, indicate an interruption of the object in the two images, suggesting spatial discontinuity.

The method proposed by Kim, Kanezaki, & Tanaka [38] evaluates the absolute distance between the original image and its response map in several directions. This method identifies discrepancies in the continuity of pixels to their neighbors, quantified by the mean absolute error $\bar{E_{abs}}$. In this paper, we propose some modifications to the approach of Kim, Kanezaki, and Tanaka [38]. For example, we introduce the function Δ(*r*′ *i*, *j*) to replace $\bar{E_{abs}}$. The authors of the original paper consider the distance λ to be constant for all cases, λ = 1. In contrast, we determine the distance λ based on the desired value of *N*_*R*_, which is treated as a hyperparameter to be optimized. For the specific region in the image considered as the center point and the corresponding region in the other image after applying different *N*_*F*_, Δ(*r*′ *i*, *j*) measures the difference between these regions. In Cartesian coordinates, Δ(*r*′ *i*, *j*) tends to be more pronounced at the most extreme points. These adjustments include the introduction of dynamic values for comparing the central image and its response map, as well as specific values to determine the desired *N*_*R*_. Similar to Shibata et al. [81], we consider the norm-$\Delta (r_{i,j}^{\prime })$ of the horizontal and vertical differences of the response map $r_{i,j}^{\prime }$ as a spatial constraint. Thus, the process can be implemented through the differential operator, defining the spatial continuity loss given by:

$$\begin{array}{c} \Delta \left(r_{i,j}^{\prime }\right)=\sum_{i=1}^{w-1}\sum_{j=1}^{h-1}||r_{i+\lambda ,j}^{\prime }-r_{i,j}^{\prime }||\lambda +||r_{i,j+\lambda }^{\prime }-r_{i,j}^{\prime }||\lambda , \end{array}$$ (2)

where λ is the distance to be determined based on the desired *N*_*R*_, *r*′ *i*, *j* refers to the response map, *r*′ *i* + λ, *j* and $r_{i,j+\lambda }^{\prime }$ represent the pixel values at positions *i*, *j* in the response map, and *w* and *h* correspond to the length and width of the image, respectively. The selection of the λ value plays a crucial role in the segmentation of structures in medical exams. Choosing a smaller λ value may result in smaller regions being neglected in the segmented exams, while a larger λ value has the potential to segment small and disconnected structures.

The proposed evaluation function *F*_*aval*_ considers the composition of *S* and $\Delta (r_{i,j}^{\prime })$. It analyzes the similarity between pixels adjacent to a given pixel, also considering the nearby neighbors. The process begins with feature extraction and culminates in the assignment of labels or categories to the images contained in the tensor [*P*_*n*_]. The tensor results from the feature extraction and normalization step and encapsulates the relevant features of the original images. After processing and normalization of [*P*_*n*_], the rectified linear activation function (ReLU) is used for normalization. This leads to a non-linearity in the outputs of the neural network layers. The evaluation function is therefore given by:

$$\begin{array}{c} F_{aval}=\left(d_{sim}\cdot S\right)+\left[d_{cont}\cdot \Delta \left(r_{i,j}^{\prime }\right)\right] \end{array}$$ (3)

where *d*_*sim*_ and *d*_*cont*_ are regularization parameters used to find the balance between fitting the data in *S* and in Δ(*r*′ *i*, *j*). They determine the penalty applied in *Faval* according to the magnitude of the model, ensuring that the fitting process is sensitive to the data. This approach helps prevent overfitting, which could lead to difficulties in generalizing to new datasets, and contributes to the model. In the work of Kim, Kanezaki, & Tanaka [38], these parameters are considered constant and both are assigned the same value so that *S* and Δ(*r*′ *i*, *j*) have no penalty in *Faval*. However, in this work, we propose to optimize the values of *d*_*sim*_ and *d*_*cont*_, which are indirectly used in the cross-entropy function given by (1), in the calculation of *S*, and in $\Delta (r_{i,j}^{\prime })$.

The data structure resulting from the permutation, with dimensions [*N* × *P*_*n*_], is submitted to the application of a one-dimensional filter after the evaluation step. This filter is represented by the red flow in Fig 4 and transforms the data structure into a tensor of dimension [*N*], in which the maximum values are highlighted. The criterion for selecting the key pixels in the one-dimensional filter is based on intensity. This criterion aims to maintain continuous structures while suppressing artifacts that could interfere with cohesion. After applying the one-dimensional filter, Remodeler 2 reconstructs the tensor, enhances the edges and boundaries of each object, and obtains the image of the partial segmentation.

The grouper aims to consolidate pixels by combining repeating values into individual elements. In this way, the labels present in the image are counted. From the one-dimensional vector obtained in the filter step, its elements are identified, classified as unique, and organized. This step results in the unification of pixels in the image, and the number of unique pixels can be quantified, corresponding to the number of structures identified in the unsupervised segmentation. To calculate *S*, the cross-entropy algorithm described by (1) is used. This process compares the data structure resulting from the permutation step with the maximum values from the filter step.

The unsupervised segmentation approach is automatically guided by two metrics: Δ(*r*′ *i*, *j*) and *S* in (3). Δ(*r*′ *i*, *j*) controls the process through mathematical operations that outline the contours of the objects or structures present in the image. At the same time, the degree of similarity between neighboring pixels is evaluated by calculating *S*. The combination of these two metrics enables the identification, delineation, and segmentation of specific regions in the image. Δ(*r*′ *i*, *j*), the calculation of *S*, and *N*_*R*_ are used as stopping criteria for the segmentation algorithm. The algorithm therefore stops when *N*_*R*_ is reached **or** when the maximum number of iterations *NI* is reached.

### Hyperparameter optimization

Hyperparameters are predefined configurations that guide the training process of a machine learning model. Unlike model parameters, which are directly adjusted by the data, hyperparameters are defined before training begins and remain constant throughout the entire process. The correct selection of these hyperparameters is essential to achieve satisfactory model performance, thereby saving time and computational resources. In this study, as shown in Fig 3 in the blue block, an optimization process is applied to determine the most effective hyperparameters, including: i) optimizer *O*_*PT*_, ii) number of convolutional filters *N*_*F*_, iii) number of convolutional layers *N*_*C*_, iv) distance λ, v) maximum number of iterations *N*_*I*_, vi) regularization rate *d*_*sim*_, vii) regularization rate *d*_*cont*_, and viii) learning rate *T*_*A*_, where $N_{F},N_{C},N_{I},\lambda \in Z^{*}$, and $d_{sim},d_{cont},T_{A}\in R^{*}$.

The choice of *O*_*PT*_ involves evaluating different algorithms to determine which are more efficient in identifying the desired *N*_*R*_. The configuration of *N*_*F*_ refers to determining the number of *N*_*C*_ in the feature extractor. The optimization of these quantities aims to minimize the evaluation function given the desired *N*_*R*_. Defining the appropriate amount of *N*_*C*_ in the architecture of the feature extractor aims to achieve optimal efficiency and capture precise details. Having too few layers may be insufficient to generate the desired features while having too many layers can lead to saturation in feature extraction [92]. The hyperparameter λ is optimized within a predefined value range. Larger values neglect smaller regions in segmented exams, and smaller values segment small and disconnected structures.

When validating the methodology, a loop with a predefined maximum *N*_*I*_ is used. The optimization of *N*_*F*_ is necessary as it aims to track the progress of *F*_*aval*_ during training, allowing comparisons between different hyperparameter configurations. Moreover, this controllable constraint allows simulating the performance of the methodology during the segmentation of brain structures. The value of *S* is used to ensure that neighboring pixels belong to the same group. However, for images with similar pixel intensities, the values of *S* can be close to zero even if the images are different. To control the optimization of *S*, the optimized value is assigned to*d*_*sim*_. To achieve this, the range of values for the optimal or optimized ratio between *S* and *N*_*R*_ must be determined. The effectiveness of the features generated by the extractor is directly linked to *T*_*A*_. Low values of *T*_*A*_ can cause the network to stagnate in local minima, while high values can cause the network to continue training even after reaching an optimal or optimized point.

### Evaluation of the proposed model

The model evaluation process consists of two stages: the first involves comparison with another established segmentation method in the literature, as illustrated in the green block of Fig 3, using the Dice Sørensen coefficient *D*_*c*_, a similarity measure commonly used in segmentation assessments [93–95]. The *D*_*c*_ provides a quantitative assessment of the quality of the segmentation generated by the proposed method in comparison to the existing approach. In the second stage, the evaluation is performed by a trained neuroradiologist who examines the segmentations produced by the proposed method.

The specialist evaluates criteria such as: i) accuracy, ii) delineation of contours, and iii) ability to identify relevant features or regions. This qualitative assessment complements the quantitative analysis and validates the efficiency of the new method. After validation, the trained model can be used as a segmentation tool for different brain CT scans and datasets, eliminating the need for further validation. In other words, neither the use of the segmentation block with another method in the green block of Fig 3 nor the evaluation block with a specialist is required.

## Results

This section presents the results of the application of the proposed method. This includes describing the employed database, optimizing the hyperparameters, performing the segmentation phase, and conducting subsequent statistical analysis. They are complemented by the illustrations in Figs 3 and 4.

The unsupervised methodology produces segmentation masks, in contrast to results generated by the CTSeg tool [82, 83], which are images with predefined labels. To ensure uniformity, we will refer to the methodology’s results as labels. This terminology choice facilitates the comparison of segments generated by this approach with those from the CTSeg tool, enabling a comprehensive assessment of similarity and confirming that the analyzed segmentation aligns with the corresponding label generated by CTSeg.

### Dataset and parameters definition

Since we addressed the Computed Tomography Quality 500 (CQ500) database, a public dataset containing 500 brain CT scans performed by various hospitals in India. This dataset includes high quality images that have been properly anonymized [25]. In addition to the images, CQ500 provides clinical reports prepared by three radiologists with 8, 12, and 20 years of experience in brain CT interpretation. The image files are structured as follows: i) patient identification, ii) study identification, and iii) exam identification, with the files in DICOM format. The exams are identified by codes starting with CQ500**CT*00x***, where ***00x*** represents the exam ID. In this work, we have decided to remove the prefix CQ500 and retain only the suffix CT*00x*.

Segmentation with a different method, as shown in Fig 3, uses the CTSeg tool [82, 83]. An acceptable threshold of *η*_*c*_ = 0.8 was set, indicating that metrics with higher values indicate greater similarity between images. Following the Pareto principle [96, 97], the selection of 100 reference exams from the set of 500 exams of CQ500 was performed. Among these 100 exams, one was selected to adjust the hyperparameters to minimize the evaluation function. For the experiments, five exams were randomly selected from the remaining 400 according to the Pareto principle. No abnormalities or findings outside the normal range were detected when analyzing these five exams.

The exams selected for the experiments are: i) CT047, which serves as a reference for hyperparameter tuning, and ii) CT042, C195, CT200, CT299, and CT418 for testing and validation. Each of these images has a resolution of *A* = 512 × *L* = 512 pixels, resulting in a total number of *N* = *A* ⋅ *L* = 262144 pixels. After preprocessing, a data structure with the dimensions [*N*_*f*_ = 256, *L* = 512, *A* = 512] is obtained. In this study, the data is transformed into images with a structure of *P*_*n*_ = 100. Each position in this structure contains a processed image, maintaining the original dimensions of *A* × *L* pixels. The proposed unsupervised segmentation method requires pre-training of the feature extractor, which is the core of the process.

Training is performed for each desired *N*_*R*_ so that the trained neural network can subsequently be used to segment other exams and validate the results obtained. In our experiments, validation was performed with the five scans CT042, CT195, CT200, CT299, and CT418. Although it is possible to use random slices of exams for segmentation, it is important to consider that brain CT scans have sequencing in the slices and share similar information between adjacent slices. A single brain CT scan can provide sufficient and representative information for neural network training due to the similarities between different slices of the same scan. This approach saves computational resources and time and simplifies the training process. By choosing to train on a single random exam with *N*_*f*_ = 256 slices, the unsupervised method proves to be an efficient and feasible strategy. This is particularly important because unsupervised methods, unlike supervised methods, do not require large amounts of data for training [98, 99].

### Optimization results

The search for optimized values for the hyperparameters controls the behavior of the feature extractor during training as well as the behavior of the functions responsible for segmentation. The hyperparameters considered include: i) optimizer *O*_*pt*_, which includes three different algorithms: Adam, RMSprop, and SGD, ii) the number of neurons per layer *N*_*F*_, ranging from [15, 150] neurons, iii) the number of convolutional layers *N*_*C*_, ranging from [1, 9] layers, iv) the distance λ, with values in the range of [1, 9], v) the maximum number of iterations *N*_*I*_, ranging from [1, 10] iterations, vi) regularization terms for similarity *d*_*sim*_ and continuity *d*_*cont*_, with values ranging from [0.1, 5] in increments of 0.1, and vii) learning rate *T*_*A*_, in the range [0.001, 0.1].

The three optimization techniques were considered due to their popularity in machine learning and neural network training [100, 101]. The ranges of hyperparameters to be optimized were selected based on the empirical knowledge of the researchers. To optimize these hyperparameters, the Optuna search tool was used [78]. A total of twelve simulations were performed for each *N*_*R*_, covering the range with $N_{R}\in Z^{*}$, 3 ≤ *N*_*R*_ ≤ 8 labels. Table 2 disposes the averages $\bar{D_{c}}$ and standard deviations *σ* for *D*_*c*_ along the twelve simulations for each *N*_*R*_ and for each class: gray matter *c*_1_, white matter *c*_2_ and skull *c*_4_. The reference segmentations considered to calculate *D*_*c*_ are from the CTSeg tool, and the calculation of *D*_*c*_ is performed by the 3D Slicer, an open-source software platform for processing and analyzing medical images [102].

**Table 2:** $\bar{D_{c}}$ and *σ* along the twelve simulations for each *N*_*R*_ and for each class *c*_1_, *c*_2_ and *c*_4_.

File	*N*_*R*_	$\bar{D_{c}}$ and *σ* of *c*_1_	$\bar{D_{c}}$ and *σ* of *c*_2_	$\bar{D_{c}}$ and *σ* of *c*_4_
CT042	3	–	–	0.80 ± 0.03
	4	–	–	0.59 ± 0.06
	5	–	–	0.55_0.02_
	6	–	0.55 ± 0.06	0.64 ± 0.03
	7	–	0.39 ± 0.05	0.69 ± 0.04
	8	–	0.40 ± 0.07	–
CT195	3	0.50 ± 0.03	–	0.74 ± 0.01
	4	0.49 ± 0.05	–	0.70 ± 0.03
	5	0.44 ± 0.05	0.25 ± 0.06	–
	6	0.57 ± 0.05	0.30 ± 0.05	0.65 ± 0.03
	7	0.57 ± 0.04	0.40 ± 0.05	0.70 ± 0.03
	8	0.45 ± 0.05	0.35 ± 0.07	–
CT200	3	0.43 ± 0.05	–	0.70 ± 0.05
	4	0.43 ± 0.03	–	0.72 ± 0.03
	5	0.42 ± 0.02	–	0.45 ± 0.06
	6	0.45 ± 0.04	0.42 ± 0.08	0.75 ± 0.04
	7	0.58 ± 0.03	0.35 ± 0.10	0.80 ± 0.02
	8	0.46 ± 0.04	–	0.45 ± 0.06
CT299	3	0.42 ± 0.05	–	–
	4	0.40 ± 0.04	–	–
	5	0.48 ± 0.03	–	–
	6	0.45 ± 0.06	0.58 ± 0.05	–
	7	0.50 ± 0.02	0.60 ± 0.06	–
	8	0.35 ± 0.06	–	–
CT418	3	0.45 ± 0.08	–	0.70 ± 0.03
	4	0.42 ± 0.09	–	0.65 ± 0.07
	5	0.40 ± 0.05	–	–
	6	0.42 ± 0.03	0.55 ± 0.05	0.72 ± 0.05
	7	0.60 ± 0.03	0.40 ± 0.05	0.82 ± 0.04
	8	0.45 ± 0.04	–	–

Note in the Table 2 the absence of some $\bar{D_{c}}$ values. These omissions are related to discrepancies between the results of the proposed segmentation model and the reference segmentation. Unsupervised methods do not guarantee identical results as they do not include training of weights and classifiers. For example, when using the segmentations of the CTSeg tool as a reference, the segmentation obtained with the proposed model may exhibit variations in brain tissues and structures. These divergences can lead to a lack of agreement compared to *D*_*c*_.

The most efficient configuration from the twelve simulations is the one with the best averaged *D*_*c*_ among the three classes *c*_1_, *c*_2_ and *c*_4_. Table 3 disposes results of the hyperparameter optimization process, displaying the most efficient configuration for each *N*_*R*_ value, along with the processing time *t* [*min*] for each optimization process. In the *N*_*R*_ column, the predefined amount of desired segmentation is given. We observe an increase in the expansion of the neural networks, both in width and depth, to find the quantity of labels. Moreover, different *T*_*A*_ values were specified for each optimizer.

**Table 3:** Hyperparameters optimized based on the predefined quantity of labels.

*O*_*pt*_	*N*_*F*_	*N*_*C*_	λ	*N*_*I*_	*d*_*sim*_	*d*_*cont*_	*T*_*A*_	*N*_*R*_	*F*_*aval*_	*t* [*min*]
RMSprop	125	5	1	9	0.2	0.1	0.02071	3	0.0240	120
SGD	55	3	1	6	1.8	0.1	0.07809	4	0.0776	22
SGD	85	1	3	6	1.8	5.0	0.06904	5	0.1666	44
RMSprop	125	3	1	10	1.4	1.9	0.00532	6	0.0932	48
SGD	45	3	1	3	0.4	3.4	0.08104	7	0.2999	100
ADAM	65	1	7	3	1.6	1.6	0.04715	8	0.2212	60

### Comparison between segmentation methods

**Fig 5:**
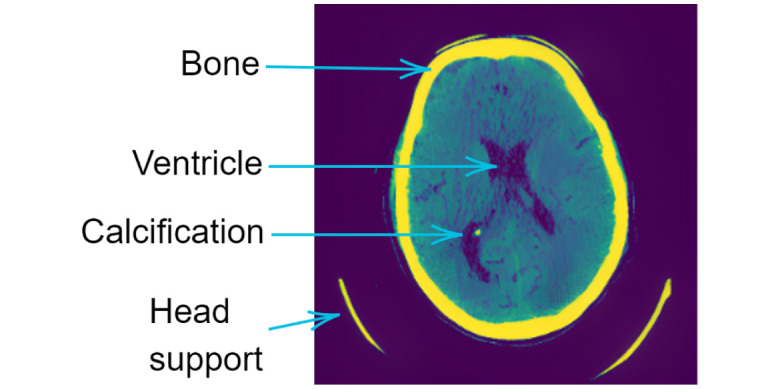
Windowing filter 40 × 80 [*HU*].

The proposed method starts with a window level of 40 [*HU*] and a window width of 80 [*HU*]. This procedure ensures the presence of tissue of interest, such as the white and gray matter, while excluding unwanted physical artifacts that may be present in the exam. However, this filter can lead to disturbances such as the presence of isolated pixels or small groups of pixels with extremely low values (salt-type noise) or extremely high values (pepper-type noise) [91, 103, 104]. These isolated points or small groups of pixels with discrepant values can distort the image information, making interpretation and clinical use more difficult. Fig 5 presents the result after applying windowing. We notice that the region containing brain tissue has salt and pepper noise. There is also differentiation of the ventricular structure, including calcification of the choroid plexus, a physiological phenomenon [105].

**Fig 6:**
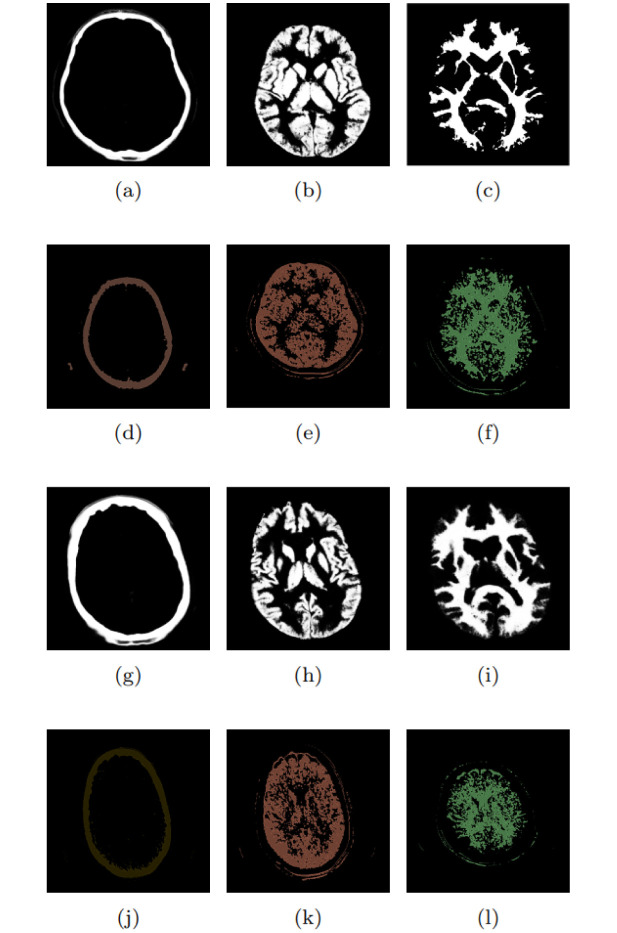
Segmentation of exams: (a) to (f) CT042 and (g) to (l) CT195.

**Fig 7:**
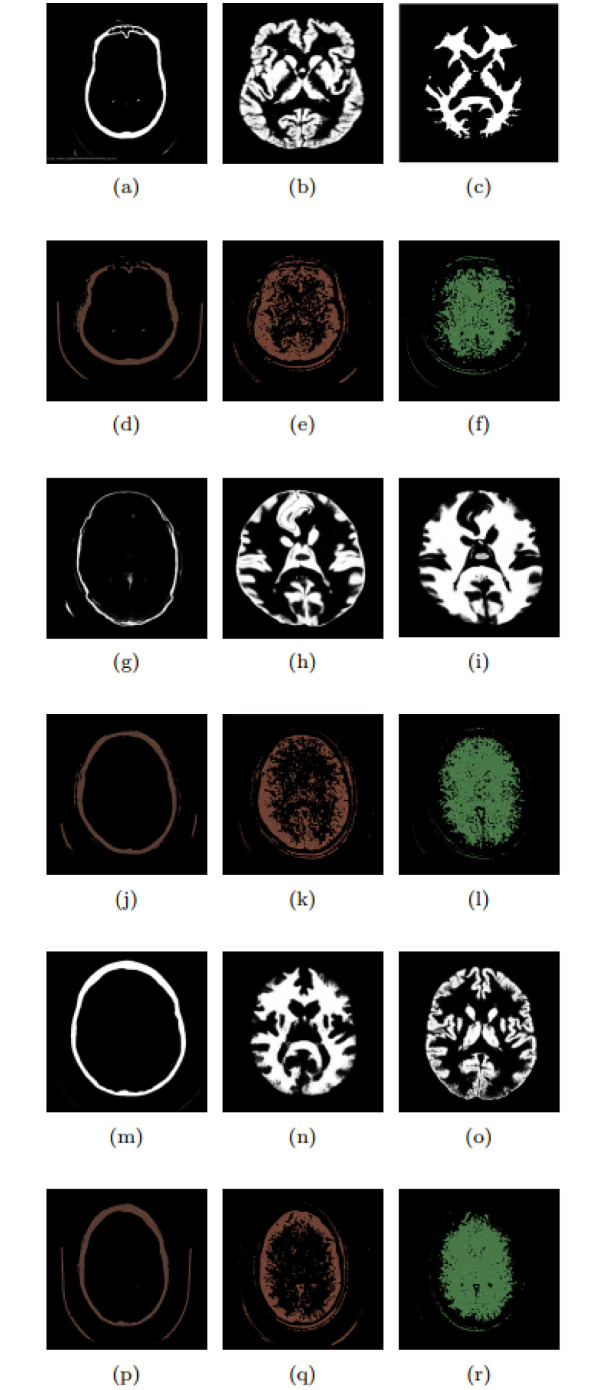
Segmentation of exams: (a) to (f) CT200, (g) to (l) CT299, and (m) to (r) CT418.

If applying the CTSeg tool, it is not necessary to specify *N*_*R*_ because the classes are automatically identified during processing and the human anatomical structures are recognized. Typically, the labels include gray matter, white matter, CSF, skull, extracranial soft tissue, and a background label that does not correspond to any of the previous labels. Three classes are used in the validation to avoid favorable bias, which have better segmentation performance compared to the CTSeg tool: i) skull, ii) gray matter, and iii) white matter. The segmentation results were obtained using both the CTSeg tool and the proposed method, which were set with the optimized hyperparameters from Table 3 and adjusted for the same *N*_*R*_ as the CTSeg tool. Figs 6 and 7 show these results for exams CT042, CT195, CT200, CT299 and CT418, respectively.

In Figs 6 and 7, the black and white images show the segmentation results obtained with the CTSeg tool, while the multicolored images show results of the proposed approach. Thus, the images are assigned to three different tissue classes: i) the first column represents the skull (bone) *c*_4_, ii) the second column corresponds to the gray matter *c*_1_, and iii) the third column represents the white matter *c*_2_. Results obtained with the optimized parameters shown in Table 3 are presented in Table 4 and compared with the approach proposed by Kim, Kanezaki, and Tanaka [38]. The evaluation of results is expressed in the form of *D*_*c*_. The reference segmentations considered to calculate *D*_*c*_ are from the CTSeg tool, and the calculation of *D*_*c*_ is performed by the 3D Slicer. The range is 0 ≤ *D*_*c*_ ≤ 1, where 0 stands for no overlap and 1 for 100% overlap.

**Table 4:** Comparison of *D*_*c*_ between the proposed method versus Kim, Kanezaki, and Tanaka’s method [38].

exam		*D*_*c*_ of *c*_1_		*D*_*c*_ of *c*_2_		*D*_*c*_ of *c*_4_	
file	*N*_*R*_	[38]	Proposal	[38]	Proposal	[38]	Proposal
CT042	3	–	–	–	–	–	0.83
	4	–	–	–	–	–	0.67
	5	0.69	–	0.31	–	**0.82**	0.57
	6	0.58	–	0.36	0.61	0.81	0.67
	7	0.73	–	0.43	0.44	0.78	0.73
	8	0.74	–	**0.60**	0.47	0.78	–
CT195	3	–	0.53	–	–	–	**0.75**
	4	–	0.48	–	–	–	0.72
	5	0.61	0.49	–	0.31	0.85	–
	6	0.51	0.59	–	0.35	0.76	0.68
	7	0.65	**0.61**	–	0.47	0.83	0.72
	8	0.67	0.49	–	0.42	0.83	–
CT200	3	–	0.48	–	–	0.84	0.74
	4	–	0.46	–	–	–	0.75
	5	0.53	0.44	0.26	–	0.86	0.50
	6	0.54	0.49	–	0.50	0.73	0.79
	7	0.55	0.61	0.40	0.45	0.81	**0.82**
	8	**0.57**	0.50	**0.44**	–	0.80	0.50
CT299	3	–	0.47	–	–	–	–
	4	–	0.44	–	–	–	–
	5	0.49	0.51	0.44	–	0.25	–
	6	0.44	0.49	0.47	0.63	0.24	–
	7	**0.50**	0.52	0.53	0.66	0.26	–
	8	0.49	0.41	**0.59**	–	0.23	–
CT418	3	–	0.53	–	–	**0.85**	0.73
	4	–	0.50	–	–	–	0.72
	5	**0.58**	0.45	0.34	–	0.83	–
	6	0.56	0.45	0.37	0.60	0.72	0.77
	7	–	0.62	0.43	0.45	0.79	0.86
	8	–	0.49	**0.50**	–	0.81	–

All exams presented in Table 4 are the results of about 256 slices after preprocessing. Overall, the values presented in Table 4 show an accuracy of over 65% for the proposed approach, compared to an accuracy of about 33% for the method of Kim, Kanezaki, and Tanaka [38]. The optimization performed resulted in a significant increase in the accuracy of Kim, Kanezaki, and Tanaka’s method [38] when using the proposed method. When analyzing the segmentation results for class *c*_1_ in the CT042 exam, it was found that the CTSeg segmentation had no significant correlation with the segmentation of the proposed method. In contrast, when comparing with the segmentation using Kim, Kanezaki, and Tanaka’s method [38], a lack of correlation was found only for *N*_*R*_ = 3 and *N*_*R*_ = 4, with 0.58 ≤ *D*_*c*_ ≤ 0.74. Considering all exams for the class *c*_1_, the proposed method reached 0.41 ≤ *D*_*c*_ ≤ 0.62, while the method of Kim, Kanezaki, and Tanaka [38] reached 0.44 ≤ *D*_*c*_ ≤ 0.74.

Except for exam CT042, segmentation with CTSeg showed an association in all other exams compared to the proposed method, including all labels of class *c*_1_. The proposed method performed better in four exams than Kim, Kanezaki, and Tanaka’s method [38], which segmented all exams but performed better in only two of them. For classes *c*_1_ and *c*_2_, the proposed method showed better results in terms of *D*_*c*_ than the approach of Kim, Kanezaki, and Tanaka [38]. The optimization provided average improvements of 4% for class *c*_2_ and 5% for class *c*_4_ compared to the method of Kim, Kanezaki, and Tanaka [38]. These results show that the proposed work with hyperparameter optimization can achieve more accurate white matter segmentation than the approach of Kim, Kanezaki, and Tanaka [38].

The white matter is the brain tissue that establishes the connections between different brain regions and plays an important role in cognitive and motor functions. Precise segmentation of white matter is essential in the imaging assessment of neurological diseases such as multiple sclerosis, dementia and brain tumors [106–108]. Regarding the overall results for the segmentation of class *c*_4_, the approach of Kim, Kanezaki and Tanaka [38] achieved values of 0.23 ≤ *D*_*c*_ ≤ 0.86, while these values for the proposed method were 0.50 ≤ *D*_*c*_ ≤ 0.86. When comparing the two methods, it can be seen that of the three classes and five exams analyzed, the proposed method outperforms in ten exams, while the method of Kim, Kanezaki and Tanaka [38] outperforms in five exams, considering the results in terms of *D*_*c*_. These results show that the proposed method segments different tissues in CT scans, generating masks similar to those of the CTSeg tool.

The optimization process proved to be efficient since there was no saturation for the class *c*_4_, indicating a satisfactory performance of *F*_*eval*_. However, when considering only the best *D*_*c*_ result for each exam in the comparison between the methods, we observed that the proposed method performs worse on the segmentations of class *c*_4_. Although the proposed method generates segmentations for 3 ≤ *N*_*R*_ ≤ 8, this does not necessarily mean that the class *c*_4_ in CTSeg will find similar segmentations for all labels generated by the proposal. The presence of the class may or may not be present in each label. When analyzing the segmentation difficulty, we found that out of the 30 segmentations performed in class *c*_4_, eleven of them have no correlation between the proposed method and the CTSeg segmentations. Moreover, in the CT299 exam, the approach of Kim, Kanezaki, and Tanaka [38] for class *c*_4_ achieved a better result with *D*_*c*_ = 0.26. These results indicate the complexity of the segmentation of the class *c*_4_ and highlight the particular challenges that the proposed method has to face compared to the approach of Kim, Kanezaki, and Tanaka [38].

A detailed analysis of the segmentation results of the CT299 exam by CTSeg, presented in Fig 7(h) and 7(i), reveals asymmetric distortions that indicate segmentation errors. These distortions are noticeable from class *c*_4_, Fig 7(g). Here, disturbances occur in the bone segment, accompanied by variations in bone thickness and a distorted morphology. Compared to the segmentation results of our approach, presented in Fig 7(j), no distortions are observed in class *c*_4_. Instead, there is bone formation in an ovoid shape as expected for the human skull. Moreover, we observed in Fig 7(k) and 7(l) we observed that the regions corresponding to classes *c*_1_ and *c*_2_ maintain the integrity of the tissue without exhibiting asymmetric distortions. This comparison shows that the segmentation performed with the proposed method can in some cases be superior to the segmentation performed with the reference method, the CTSeg tool.

**Fig 8:**
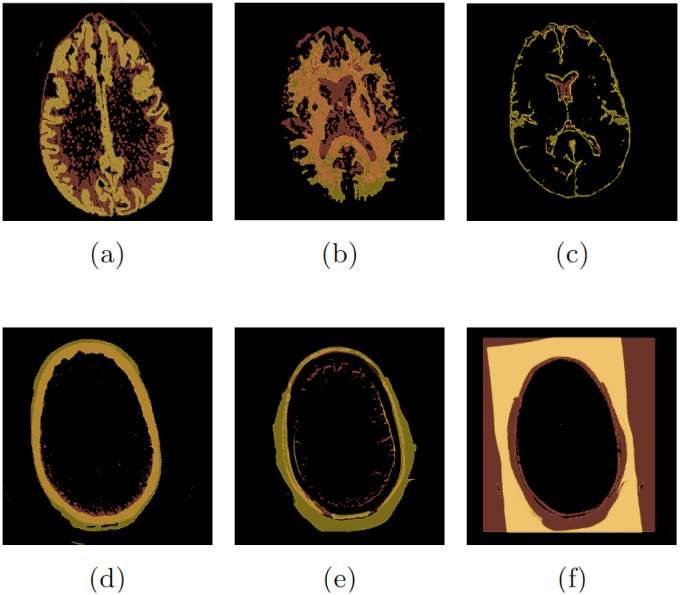
Segmentation overlap of the CT195 exam: (a) *c*_1_, (b) *c*_2_, (c) *c*_3_, (d) *c*_4_, (e) *c*_5_ e (f) *c*_6_.

Fig 8 shows the overlap of the segmentation of the CT195 exam performed by the CTSeg tool and the segmentation obtained by the proposed method. By relating the six labels used by CTSeg to the labels obtained by the proposed method, we were able to identify overlap in classes *c*_1_, *c*_2_, and *c*_4_, as well as in certain classes: CSF class *c*_3_, soft tissue class *c*_5_, and background class *c*_6_. Fig 8(a) to 8(f) show a partial correspondence between the segmentations of the CTSeg tool and the proposed method.

For the class segmentation *c*_1_ and *c*_2_, shown in Fig 8(a) and 8(b), respectively, the result in the deep shade is the entire set, while the segmentation by the CTSeg tool in the light shade is the correct subset. In contrast, for the class segmentation *c*_3_, *c*_4_, and *c*_5_, shown in Fig 8(c)–8(e), respectively, there is an intersection, where the segmentation by the CTSeg tool in the light shade is the set and the segmentation by the proposed method in the deep shade is the correct subset. Finally, for the segmentation of class *c*_6_, shown in Fig 8(f), the CTSeg tool incorrectly considered classes *c*_4_, *c*_5_, and *c*_6_, resulting in an inaccurate segmentation, while the proposed method performed the segmentation correctly.

Results presented in Fig 8 underscore the importance of precise segmentation in medical imaging. The reliability of the information obtained from these exams is inextricably linked to the quality of the segmentation, which has a direct impact on diagnoses and clinical decisions. Identifying these symmetric or asymmetric distortions in the segmentation process emphasizes the need to review and optimize procedures. This is crucial to ensure the accuracy and reliability of diagnostic results.

### Comparison of the cranial volumetry

Volumetry of head structures is crucial in neurological assessments, especially in neurodegenerative diseases and brain tumors (both in surgical planning and post-treatment monitoring) [109]. The calculation process involves the segmentation of images to identify and delineate a specific structure (e.g. the skull or an intracranial region), resulting in the creation of a three-dimensional model. The volume is determined by multiplying the segmentation area by the slice thickness to obtain the volume for each slice. The volumes of all slices are then summed to determine the total volume of a particular structure. Three-dimensional reconstruction software such as 3D Slicer [102] is used to create volumetric models from segmented images.

Cranial volumetry using segmentations obtained with the CTSeg tool is compared with the proposed method. The segmentations from both approaches were used in the 3D Slicer platform to model the three-dimensional system and calculate the cranial volumes in selected exams with the highest *D*_*c*_ values from Table 4. Results of this validation are shown in Table 5, where $V_{c_{4}}$ represents the cranial volumetry values for class *c*_4_. It can be observed that the values of $V_{c_{4}}$ are higher in 62.5% of the cases with the proposed method. This is due to the application of post-processing techniques by the CTSeg tool, which improves the segmentation contours [82, 83], reducing the value of $V_{c_{4}}$.

**Table 5:** Comparison of cranial volumetry values between the proposed method versus CTSeg.

exam		$V_{c_{4}}$ [*cm*^3^]		$\bar{V_{c_{4}}}$ [*cm*^3^]	$\sigma^{V_{c_{4}}}$ [%]
file	*N*_*R*_	CTSeg	Proposal		
CT042	3	600.85	679.10	639.97	11.5
	5	600.85	664.67	632.76	9.6
CT195	3	789.14	756.35	772.74	-4.3
	5	789.14	712.20	750.67	-10.8
CT200	5	739.56	762.06	750.81	2.9
	6	739.56	992.81	866.18	25.5
CT418	3	596.89	624.79	610.84	4.4
	7	596.89	593.89	595.39	-0.5

**Fig 9:**
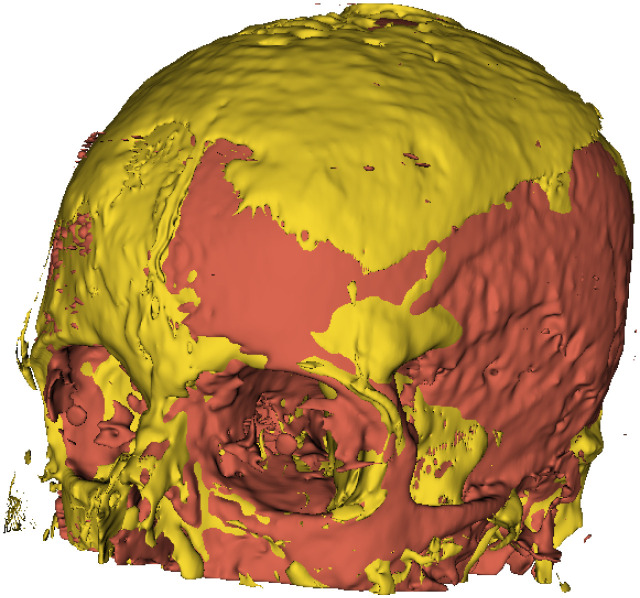
Cranial volumetry for class *c*_4_ in the CT042 exam.

In Table 5, $\bar{V_{c_{4}}}$ represents the average of the values, while $\sigma^{V_{c_{4}}}$ indicates the percentage error between the values determined with the two methods, using the values determined with the CTSeg tool as a reference. Fig 9 shows a visualization of the cranial volume of class *c*_4_ in the CT042 exam with *N*_*R*_ = 3, where the segmentation with the proposed method is shown in a deep shade and the CTSeg tool in a light shade. As observed in Table 5, the volume resulting from the segmentation of the class *c*_4_ by the proposed method is larger than the volume obtained by the CTSeg tool. Fig 9 is intended to facilitate visualization in the three-dimensional plane, since the segmentations are performed in a two-dimensional plane. By organizing the data, each segmentation is integrated into a resulting matrix that can be interpreted as a three-dimensional volume, enabling projection and subsequent visualization.

By quantifying the volume of segmented structures in a given patient, it is possible to compare them with other patients in the same database, which facilitates the identification of discrepant values. Our approach enables the use of the automatic segmentation model for screening and prioritization of suspected cases, accelerating the diagnosis of critical diseases at an early stage. Moreover, the efficiency and cost-effectiveness of the proposed method are remarkable. Since it is an unsupervised model, the fast and efficient computational power reduces the costs associated with data processing and makes the technology more accessible, especially in resource-constrained contexts. Although CT is more accessible than MRI, deep learning models for CT segmentation are less common than those developed for MRI. This work is an important step towards reducing this disparity in clinical practice.

### Evaluation and validation of the proposed methodology

The validation of the proposed method for unsupervised structural segmentation in brain computed tomography is conducted through the analysis of results by expert physicians in intracranial imaging, encompassing a detailed evaluation of all outcomes. These processes ensure the efficiency, reliability, and clinical relevance of the method, demonstrating its segmentation capability and utility for diagnosis and treatment planning in clinical settings.

#### Validation by medical specialists

Results were subjected to expert analysis, revealing that the comparative analysis of CT brain segmentation offers valuable insights into process efficiency and reveals relevant aspects for clinical interpretation. Within the CQ500 dataset [25], we observed a diverse array of supports for fixing the patient’s head, suggesting the use of different CT scanners. This diversity is beneficial as it introduces variability into the proposed method, enabling more robust assessments across different CT scanner configurations. The evaluation included several variables, from the influence of device diversity to optimization with the configuration of the number of labels, with a range of values of 3 ≤ *N*_*R*_ ≤ 8, which was considered an unnecessary interval in some exams. This range overlaps with most brain tissues, considering the Hounsfield scale, and provides less visualization and detail of the anatomical structures. The appropriate choice of this range is necessary to avoid loss of information or excessive detail that could affect the results, ensuring coherent segmentation of tissues and structures.

**Fig 10:**
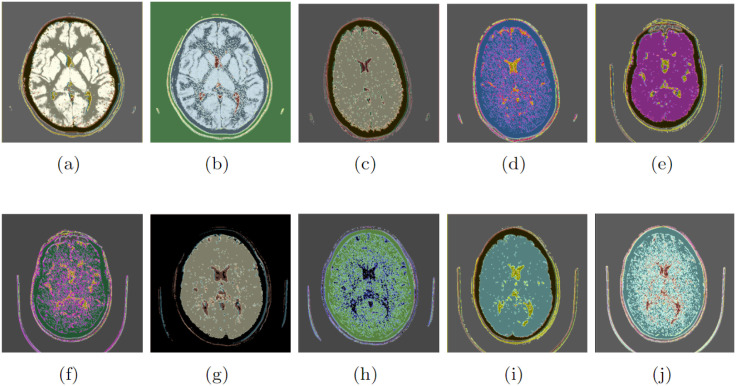
Segmentation using the proposed method in exams: (a) CT042 *N*_*R*_ = 7, (b) CT042 *N*_*R*_ = 8, (c) CT195 *N*_*R*_ = 7, (d) CT195 *N*_*R*_ = 8, (e) CT200 *N*_*R*_ = 7, (f) CT200 *N*_*R*_ = 8, (g) CT299 *N*_*R*_ = 7, (h) CT299 *N*_*R*_ = 8, (i) CT418 *N*_*R*_ = 7, and (j) CT418 *N*_*R*_ = 8.

The experts discussed the visual analysis of the results and emphasized the importance of the choice of *N*_*R*_ in the segmentation. The preference for *N*_*R*_ = 6 was described by the experts as visually appealing since it allows for differentiation between white and gray matter. It was suggested to reduce the labeling range to 3 ≤ *N*_*R*_ ≤ 6, supported by the observation that this range provides more clarity and anatomical distinctiveness. Results of the CT042, CT195, CT200, CT299, and CT418 exams for *N*_*R*_ = 7 and *N*_*R*_ = 8, shown in Fig 10, confirmed the observations for reducing the *N*_*R*_ range. Segmentation with *N*_*R*_ = 6 provided improved detail and delineation of anatomical structures. When analyzing configurations with *N*_*R*_ = 7 and *N*_*R*_ = 8 in Fig 10, a combination of different structures, such as fat and air, is observed. This phenomenon can be explained by the nature of the Hounsfield scale, which makes segmentation more difficult as the number of labels increases. In solid regions, especially at *N*_*R*_ = 8, increased speckle is observed.

The sequential analysis of the quantity of *N*_*R*_ applied to the same exam, such as the example of CT195 exam in Fig 8, shows that the proposed method can separate or group tissues according to the desired quantity. For segmentations with *N*_*R*_ = 6, we observed clarity and definition of regions, indicating the efficiency of the proposed method in delineating anatomical structures. When evaluating the CTSeg tool for CT299 exam, from Fig 7(g) to 7(i) compared to the proposed method from Fig 7(j) to 7(l), we observed asymmetric deformations in the segmentations, which casts doubt on the reliability of the reference tool. The CTSeg tool is widely used in various medical imaging centers. On the other hand, the proposed method serves as an alternative solution that represents a significant advance in brain CT segmentation and promotes improvements in some cases where the widely used method faces challenges.

#### Evaluation of results

The study presents an unsupervised methodology for brain CT segmentation, contrasting the results with the supervised method CTSeg, and utilizes the CQ500 database for validation. The generated segmentation is compared with the CTSeg tool, using the Dice coefficient *D*_*c*_ as a similarity metric. The ability of the proposed method to produce precise segmentation masks comparable to those generated by CTSeg, with a focus on the accurate identification of brain tissues, is highlighted. Additionally, a comparison was conducted between the method of Kim, Kanezaki, and Tanaka [38] and the proposed method, demonstrating segmentation accuracy exceeding 65% for the proposed method, in contrast to approximately 33% for the method of Kim, Kanezaki, and Tanaka [38]. Analyses focused on *D*_*c*_, indicating significant improvements in the results obtained by the proposed method, particularly for segmentations of gray and white matter classes, with optimizations resulting in increased segmentation accuracy.

These results suggest that the optimization of hyperparameters has contributed to enhancing the segmentation accuracy, presenting an efficient strategy for the treatment of brain CT images. Furthermore, the results indicate the effectiveness of the proposed method not only in quantitative terms, with improvements in *D*_*c*_ and segmentation accuracy but also qualitatively, by the ability to accurately segment different brain tissues compared to the CTSeg method. Detailed analysis of *D*_*c*_ indicates that, for certain classes and examinations, the proposed method outperformed CTSeg, highlighting its clinical relevance and potential application in medical practice, particularly for the precise assessment of neurological diseases through CT. This advancement in unsupervised segmentation of brain CT images may facilitate faster and more accurate diagnoses, providing a valuable tool for healthcare professionals in the evaluation of brain conditions.

### Discussion

Results obtained with the proposed method involve the unsupervised segmentation of brain CT scans, specifically applied to CQ500 database. Hyperparameter tuning was performed using optimization techniques available in the Optuna tool [78]. The experiments yielded varying Dice coefficients *D*_*c*_ for different tissue classes. These results were compared with segmentations obtained using both the CTSeg tool and the method proposed by Kim, Kanezaki, and Tanaka [38]. The comparison revealed convergence in values, with some cases favoring our proposed method. These findings align with existing literature studies [110–112] that employ segmentation techniques based on supervised models. Notably, our unsupervised model holds great relevance as it does not require large amounts of data for training, thus alleviating the computational cost in the training phase. Comparative observations between the proposed method and the CTSeg tool showed similarities in several cases. This is particularly relevant considering the widespread use of the CTSeg tool in both research and clinical settings [82, 83].

In most comparisons with Kim, Kanezaki, and Tanaka’s approach [38], our proposed method consistently outperformed. Kim, Kanezaki, and Tanaka’s method was not originally designed for medical images in the DICOM format. Regarding Dice coefficients *D*_*c*_ values, in certain cases, it was not possible to establish a direct correspondence between the labels generated with the proposed method and those obtained with the CTSeg tool. However, this lack of correspondence does not necessarily reflect the quality of the segmentation by the proposed method. The calculation of the *D*_*c*_ value is performed by 3D Slicer [102], which uses the segmentation from the CTSeg tool as a reference, including post-processing to enhance contours, reduce volume, and sometimes generate asymmetric distortions in segmentations.

This study contributes to the optimization of hyperparameters in brain CT segmentation, the innovative use of unsupervised deep neural networks, and the creation of an adjustable evaluation function for different label sets. Despite the remaining challenges in the proposed method, such as the difficulty in correlating the generated labels with the predefined labels, the observed improvements in some anatomical regions compared to existing methods highlight the relevance for clinical practice. This difficulty in correlating the labels may indicate the need for future studies on innovative data post-processing techniques, and the successes may indicate the ability of the proposed method to be applied to different types of segmentation.

Manual segmentation by trained experts is still considered the gold standard for identifying brain structures. However, this approach has its limitations, such as high costs and time-consuming manual effort, which makes it impractical for processing large amounts of data. It is also prone to intraobserver and interobserver variability. Although the segmentations generated by the unsupervised automatic model are limited to certain structures, they can serve as a basis for subsequent validation by experts, which significantly reduces the manual workload. If this model is successfully validated and integrated into real-time radiology workflows, it has the potential to become a valuable tool for screening and prioritizing exams in the radiologist’s routine.

Rapid interpretation of abnormal brain CT scans can improve patient care. The unsupervised approach does not require additional time because it does not require manual data annotation for training, as is common with supervised methods. Results of the available data and the identification of patterns or structures do not depend on manual labeling of individual inputs. Unsupervised learning can reveal non-obvious insights and patterns in the data, facilitating the identification of intrinsic relationships between variables or specific data features.

When applying unsupervised neural networks to real and previously unaudited clinical datasets, challenges arise such as variability in image quality: resolution, noise, illumination, and artifacts, which affect the network’s learning capacity and generalization. Artifacts introduced during image acquisition, processing, or transmission can distort information, while anatomical variations among patients hinder pattern identification [113]. Obtaining quality training data can also be challenging. Strategies such as data preprocessing, data augmentation, and involvement of clinical experts are necessary to address these limitations and ensure interpretability and clinical relevance of the results.

In detailed analysis of the use of automated methods for medical image segmentation in surgical and medical practices indicates both benefits and challenges. Automation offers advantages such as efficiency and accuracy in medical image interpretation but faces limitations such as dependence on data quality and the need for confidence in the results. Conversely, manual segmentation, while precise, is time-consuming and subject to interobserver and intraobserver variations. The combination of automated segmentation and human review emerges as a balanced solution, allowing validation and adjustments by experts, ensuring precise and reliable clinical decisions [114–116].

The field of medical image segmentation is rapidly advancing, with a emphasis on the use of deep learning techniques to enhance segmentation accuracy. Research demonstrates that the application of Convolutional Neural Networks (CNNs) in unsupervised scenarios yields promising results, suggesting even greater potential when combined with supervised and semi-supervised approaches. This integration aims to more efficiently capture the inherent complexities of medical images, thereby enhancing diagnostic accuracy and treatment personalization. Furthermore, the incorporation of multimodal data from various imaging modalities such as MRI, CT, and ultrasound is recognized as a necessary advancement. The combination of these diverse data sources promises to enrich segmentation models, increasing their robustness and precision by providing a more comprehensive and detailed view of the clinical aspects to be analyzed.

Another important direction for the future of medical image segmentation is the development of solutions capable of operating in real-time, especially in clinical contexts where quick decisions are crucial. The ability to perform precise segmentations instantly could revolutionize surgical and diagnostic procedures by enabling immediate interventions based on detailed and reliable information. This implies challenges both in terms of developing highly efficient algorithms and in advancing hardware infrastructure to ensure the feasibility of these technologies in clinical environments. The future of medical image segmentation focuses on enhancing accuracy through deep learning, exploring the richness of multimodal data, and implementing real-time segmentations, promising significant transformations in the healthcare field.

## Conclusion

This work developed an unsupervised segmentation method for brain CT based on the approach of Kim, Kanezaki, and Tanaka [38]. The objectives were achieved by implementing a DNN architecture to segment intracranial structures without relying on pre-existing labels, manual annotation, or supervision. Three training techniques were compared, using optimization to find hyperparameters and determine the number of segmentation masks. The method was evaluated by experts and compared with other tools. The main hypothesis was confirmed, demonstrating efficiency in training the neural network on a single random exam, reducing resources, training time, and costs, and easing the workload of the medical expert.

Compared to the approach of Kim, Kanezaki, and Tanaka, our method showed an accuracy exceeding 65%. Results indicated superior performance in white matter segmentation and similar or superior outcomes compared to the CTSeg tool in cranial volumetry. Experts recommended the range 3 ≤ *N*_*R*_ ≤ 6 to achieve visually enhanced results. This study contributes significantly to improving the accuracy of brain CT segmentation, which has promising implications in research and clinical settings. It presents a simplified and accessible approach that has the potential to facilitate early detection of abnormal scans, thereby improving patient care.
